# Right-sided hemiagenesis of the thyroid lobe and isthmus: A case report

**DOI:** 10.4103/0971-3026.40958

**Published:** 2008-11

**Authors:** Prabhat Kumar Tiwari, M Baxi, J Baxi, D Koirala

**Affiliations:** Department of Radiology, Manipal Teaching Hospital, PO Box: 341, Phulbari, Pokhara, Nepal; 1 Department of Surgery, Manipal Teaching Hospital, PO Box: 341, Phulbari, Pokhara, Nepal

**Keywords:** Congenital anomalies, hemiagenesis, thyroid

## Abstract

Unilateral or bilateral hypoplasia or agenesis of one or both thyroid lobes, with or without isthmic agenesis, is a rare developmental anomaly. Hemiagenesis of the left lobe is far commoner than of the right. Clinically, these patients may be euthyroid, hyperthyroid, or hypothyroid. Ultrasonography is usually able to diagnose this condition easily, as we demonstrate in this case report of a 37-year-old lady with an incidentally detected thyroid nodule who was found to have hemiagenesis of the right lobe and isthmus.

Developmental anomalies of the thyroid are rare. They are usually a result of abnormal descent rather than of abnormal development. Hemiagenesis of either lobe, with or without agenesis of the isthmus, is very rare, with a prevalence rate of around 0.2%[1,2] in asymptomatic children. The left side is the most commonly involved in hemiagenesis (80%). The isthmus may be absent in 40–50% of cases.[2,3] Rarely, right-sided hemiagenesis may be encountered. The disorder is more common in females, the male: female ratio being 3:1. Thyroid function may be altered in 38–47% of patients.[3] USG plays an important role in diagnosis.

We present a rare case of hemiagenesis of the right lobe and isthmus of the thyroid.

## Case Report

A 37-year-old lady presented with bilateral cyclical mastalgia and benign breast disease, which was confirmed by USG and fine needle aspiration cytology. On clinical examination, there was an enlarged left lobe; a solitary thyroid nodule, measuring 1.5 × 1.0 cm in size was also found. The right lobe could not be felt. There was no past history of surgery in the region of the neck. 

**Figure 1:**
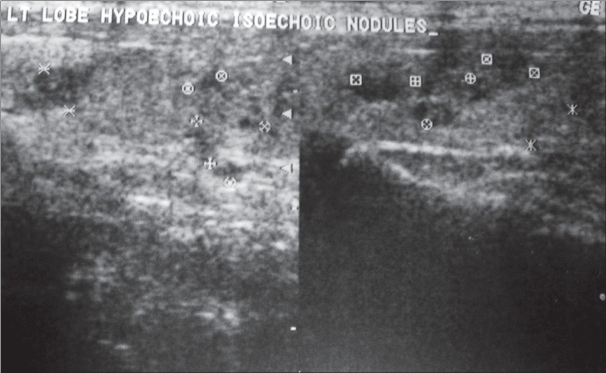
Transverse USG image shows multiple nodules in the left thyroid gland

**Figure 2:**
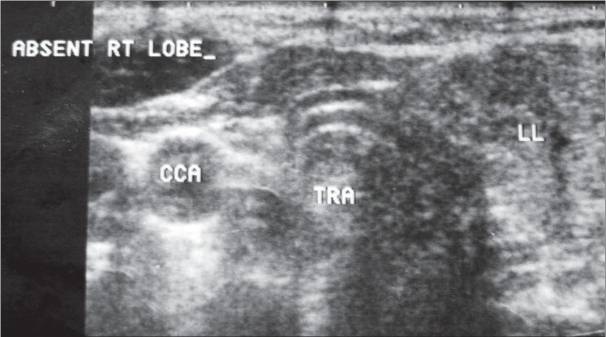
Transverse USG image shows agenesis of the right lobe

**Figure 3:**
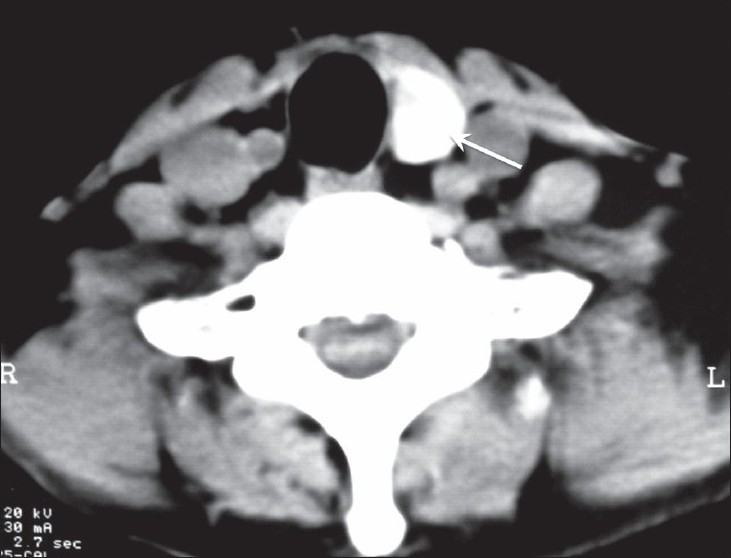
CT scan of the neck shows absence of the right lobe of the thyroid. The left lobe is well seen (arrow)

Free T_3_, free T_4_, and TSH on two occasions suggested a biochemically euthyroid state. USG showed multiple hypoechoic to isoechoic heterogeneous nodules [Figure 1] in the enlarged left lobe, the largest being 6.6 mm in diameter. Doppler showed increased vascularity in the left lobe. The isthmus and right lobe were not visualized [Figure 2]. CT scan of the neck and thyroid confirmed the agenetic right lobe and isthmus [Figure 3]. Fine needle cytology of the thyroid nodule showed a colloid nodule with cystic degeneration and hemorrhage.

The patient was reassured and was prescribed vitamin E supplementation for her mastalgia. Due to the mild symptoms and the increased vascularity of the left lobe, and in view of the possibility of developing hypothyroidism or hyperthyroidism in the future, 3-monthly follow-up with thyroid function tests and USG was advised.

## Discussion

Congenital thyroid anomalies are rare. They may be related to abnormal descent of the thyroid gland or to structural abnormalities in thyroid development, such as hypoplasia or hemiagenesis with or without isthmic agenesis.

A literature review in the year 2000 documented 256 cases of hemiagenesis.[4] We could find 51 new cases published between 2000 and 2007 in the English literature. Left-sided hemiagenesis is far commoner than right-sided hemiagenesis,[1,2,5–10] with a left : right ratio of 4:1. The prevalence of this disorder, as documented in 2845 Belgian school children who were screened by USG for congenital thyroid anomalies, was found to be 0.2%.[11]

Though the etiology of hemiagenesis is not clearly known and most of the cases are sporadic, a few may be familial and there may be a genetic predisposition.[12–16] Although patients may have a normal thyroid lobe with euthyroidism,[1,3] both hypothyroidism[13,14] and hyperthyroidism,[4,16–18] are known to occur. Other anomalies such as benign euthyroid adenoma,[19] thyroiditis,[20] multinodular goiter,[1,2,8,21] papillary carcinoma,[7,9,22] and primary hyperparathyroidism[5] have also been reported. Our patient had a euthyroid colloid nodule. The associated cyclical mastalgia and bilateral fibroadenosis were most likely coincidental findings.

As in our case, most patients with hemiagenesis are diagnosed incidentally.[1,2,8,11] USG is a useful modality to detect this anomaly. In endemic zones, there may be a high incidence of adenomatous nodules and colloid cysts in the single lobe. Malignancy always remains a cause for concern. Though a CT scan was performed in our patient, other modalities may not always be required to confirm the diagnosis of hemiagenesis, USG usually being diagnostic.[8,11,23–26]
